# Dialogue pathways and narrative analysis in health communication within the social media environment: an empirical study based on user behavior—a case study of China

**DOI:** 10.3389/fpubh.2025.1649120

**Published:** 2025-09-05

**Authors:** Xinke Wang, Xinchen Leng

**Affiliations:** ^1^School of Journalism and Cultural Communication, Zhongnan University of Economics and Law, Wuhan, China; ^2^Monash Business School, Clayton Campus, Melbourne, VIC, Australia

**Keywords:** dialogue path, emotional narrative, engagement, health communication, narrative analysis, social media, user health behavior

## Abstract

**Background:**

Social media has transformed health communication into a dynamic and interactive process, shifting from one-way dissemination by experts to user-driven content creation and sharing. However, this openness also facilitates the spread of misinformation, which poses a threat to public health behaviors. While prior research has explored dialogue paths and narrative analysis independently, there remain gaps in understanding their interactive effects and the contextual heterogeneity-such as platforms, user groups, and emotions-on health behaviors. This study addresses these gaps within China’s social media landscape.

**Methods:**

A multi-method approach was employed.

**Data collection:**

Between January 2023 and December 2024, over 50,000 data points related to health communication were collected from prominent Chinese social media platforms, including Weibo, WeChat, Xiaohongshu, and Douyin. From this comprehensive dataset, a subsample of 300 valid user cases was identified for structural equation modeling (SEM), to ensure statistical adequacy and model robustness.

**Survey:**

300 valid questionnaires assessed user perceptions and behaviors regarding health information.

**Analysis:**

Drawing upon health communication theory and narrative persuasion frameworks, a structural equation modeling (SEM) approach was employed to test the proposed conceptual model. The SEM analysis comprised two essential stages: (1) the measurement model stage, where the reliability and validity of latent constructs were evaluated through confirmatory factor analysis (CFA), including assessments of convergent and discriminant validity; and (2) the structural model stage, which examined the hypothesized relationships among dialogue path, narrative structure, engagement, and health behavior outcomes.

**Results:**

The results of the structural equation modeling (SEM) indicate that both dialogue pathways and narrative strategies significantly influence users’ health information behaviors. Specifically, dialogue depth exhibited a strong positive effect on information sharing behavior, while narrative consistency was significantly related to feedback intention. The measurement model confirmed good reliability and validity, with all factor loadings exceeding 0.7 and composite reliabilities surpassing 0.8. Normality tests indicated acceptable skewness and kurtosis for all observed variables. Furthermore, multi-group analysis revealed that platform type moderates the strength of these relationships, with Weibo users demonstrating more emotionally driven interaction patterns compared to WeChat users.

**Conclusion:**

This study identifies dialogue coherence, emotional narrative structure, and user engagement as significant predictors of health-related behavioral intentions within social media contexts. Notably, engagement serves as a crucial mediating variable that links both narrative and dialogic features to behavioral outcomes. These findings enhance our understanding of the mechanisms that underpin digital health communication, specifically by elucidating how narrative processes and dialogue influence user behavior. Practically, the results indicate that health communication initiatives on social media can be improved by integrating emotionally resonant narratives and ensuring coherence in dialogic exchanges, both of which are essential for fostering positive behavioral change. By analyzing dialogic interaction and narrative structure within a unified Structural Equation Modeling (SEM) framework, this research underscores the importance of considering storytelling elements alongside interactive features, thus providing a more comprehensive perspective on how users engage with and are influenced in digital health environments.

## 1 Introduction

In this era of social digitization and networking integration, social media enables individuals to share their daily lives and discover various new phenomena, subtly shaping societal values. Digital technology and the Internet have rendered health information less structured and accelerated its dissemination. People are no longer constrained by time and space; health information can now circulate across any platform or group. Unlike the past, where communication was predominantly one-way, led by medical institutions or experts, the current landscape fosters continuous dialogue that facilitates content sharing and interaction. With the rise of platforms such as Weibo, WeChat, Facebook, and Instagram, individuals have transitioned from passive recipients of information to active producers, becoming nodes of information dissemination rather than mere listeners. This user interaction generates a substantial amount of fragmented and dispersed information (1), which is subsequently reconstructed and localized to create content that reflects both the platform’s characteristics and user contributions, ultimately enhancing the symbiotic relationship between platforms and users. However, the very openness and accessibility of these platforms have also led to the proliferation of false information and misleading health content, posing a direct threat to public health behaviors (2). The widespread sharing of non-authoritative health information, particularly during public health emergencies or other serious health issues, tends to heighten anxiety and result in poor health decisions. Therefore, it is crucial to immediately examine the transmission channels of social media, identify credible sources of information, and understand how this information transmission influences individual behavioral choices.

Previous research has provided important insights into the roles of narrative strategies (3) in health communication. However, these studies are often limited in scope, focusing either on static, one-dimensional frameworks or isolating dialogue and narrative effects without considering their dynamic interplay within real social media environments. Furthermore, most empirical studies lack behaviorally grounded models based on actual user interactions across diverse platforms. Health narrative studies, such as those by Korda and Itani (4), primarily emphasize emotional resonance, while others, like Thon and Jucks (5), highlight credibility or empathy. Yet, these works largely rely on artificial experimental data or self-reports, lacking behavioral grounding and ecological validity. They fail to reflect the temporal, interactive, and co-constructed nature of health communication in digital spaces. Antonio et al. (6) argued that it is essential to consider the combination of regulatory mechanisms—dialogue approaches and narrative approaches—to understand their significance for the behavior of individuals seeking health information within groups. These groups rely heavily on informal communication and are particularly interested in emotional stories, underscoring the necessity of fully understanding how dialogue and narrative influence behavior through their roles in the current context.

To overcome these limitations, the present study adopts an integrated and empirically informed perspective, aiming to connect dialogue mechanisms and narrative strategies within the context of digital health communication. Rather than examining these dimensions separately, we view dialogue and narrative as interrelated mechanisms: the dialogue path shapes the flow and quality of user interactions, while the narrative structure frames health information and conveys emotional meaning. Drawing on social interaction theory, narrative persuasion theory, and the uses and gratifications approach, this research establishes a comprehensive framework for understanding how these mechanisms jointly influence user behavior across multiple platforms. This study addresses several key questions: First, it examines the independent effects of dialogue and narrative mechanisms on user behaviors such as information seeking, sharing, and providing feedback. Second, it explores the heterogeneity of these effects across user groups, emotional contexts, and different platform environments. Third, it considers how these findings can inform the development of health communication strategies that are both contextually adaptive and inclusive, thereby supporting information equity in digital settings. By grounding the analysis in real user behavior and considering platform-specific dynamics, this approach not only extends theoretical understanding but also provides practical guidance for designing more effective digital health interventions.

1. What are the independent effects of dialogue mechanism and narrative mechanism on users’ information seeking, sharing and feedback behaviors?
2. These are heterogeneous manifestations across different platforms, user groups and emotional contexts?
3. Develop environmentally sensitive and inclusive health communication strategies to promote information equity?

## 2 Reviews

Social media now plays a central role in the circulation of health and wellness information due to its interactive and dialogic nature as well as its capacity for rapid dissemination. These characteristics have made platforms such as WeChat, Sina Weibo, and Xiaohongshu important venues for public health communication and user engagement. To better understand how individuals interpret and act upon health messages in these digital spaces, this study draws on Social Constructivism as its theoretical foundation. From this perspective, knowledge and behavioral intentions are shaped through ongoing social interaction and communication (7, 8). Within this framework, dialogue between users facilitates the shared construction of meaning; narrative elements provide the cognitive and emotional context for interpretation, and user behavior emerges as a product of these socially mediated processes and perceived group norms. By considering these interrelated aspects, the study presents a model that captures how the dialogic and narrative features of social media contribute to health-related behavioral responses. Antonio et al. (6) argued that social media platforms provide a convenient and rapid means for individuals to access health information. Many individuals with specific health information needs tend to select particular platforms for discussing their health status, seeking treatment, or sharing feedback. Furthermore, the interactive nature of social media encourages users to engage in discussions regarding health issues, facilitating in-depth dialogue and comprehension on these platforms. This engagement allows more individuals to become familiar with the health information they possess or seek. Throughout the process of learning and understanding, health information can be continuously disseminated. The aforementioned research indicates that these dialogue channels are complex and dynamic. However, Smailhodzic et al. (9) argued in their review of social media and medical service systems that information on social media is decentralized and occurs within specific contexts. This suggests that the effectiveness of health communication is influenced by both social contextual factors and platform structural factors. Vraga and Bode (10) proposed that the error correction process for false health information should be viewed as a model research method of collective dialogue, which is also an important consideration when exploring the interactive model of health communication.

Information overload can lead to psychological discomfort, causing individuals to hesitate in their pursuit of information (11). Southwell and Thorson (12) elaborated on this phenomenon through their information processing bias model, which explains why users selectively engage with the vast amount of information available on social media. Heldman et al. (13) argued that for health organizations to be genuinely social, they must utilize social media to foster dialogues that reflect the dynamic, interactive, and co-creative behaviors of users, which is crucial for effective health communication strategies. The findings of this study indicate that social media not only transforms the dissemination of health information but also presents varied health messages that can influence individuals’ actions. Furthermore, Park et al. (14) employed social network analysis to reveal that the dialogue pathways within social media exhibit a center-periphery structure, wherein a limited number of influential users significantly accelerate the speed and breadth of information diffusion. Narrative analysis serves as a methodological framework for understanding how storytelling shapes representations and communication, thereby elucidating narratives. In the realm of social health communication, narrative analysis can yield valuable insights into the behaviors of individuals and groups engaged in health communication. This study aims to examine users’ self-positioning and roles in health-related posts on social media platforms, while also inferring the various narrative sources of health information involved in online health communication (15). As articulated by Chou et al. (16), it is essential to fully leverage the power of narrative in health communication processes. Emotional narratives tend to elicit greater empathy and engagement from users, particularly in discussions surrounding critical issues such as cancer and mental health. Furthermore, Bálint and Bilandzic (17) assert that the impact of media narratives in health communication depends not only on the attractiveness and structure of the story but also on users’ ability to immerse themselves in the narrative and find personal resonance within it. These factors directly influence the generation of health behaviors. The diversity of narrative types is a significant determinant of effective health communication. Jia and Qi (18) introduced a clustering model that suggests users’ willingness to share health narratives on social platforms is influenced by the strength of their social network connections, the perceived value of the content, and the level of recognition it receives. Additionally, narrative style plays a crucial role in how health messages are conveyed and perceived. Health behaviors on social media are significantly influenced by user interactions. Utilizing text classification, sentiment analysis, and topic modeling technologies on Weibo, Zhang et al. (19) identified that the identity association between users and patients shapes their discourse content, attitudes, and modes. It is essential to consider the context of user discourse and the relationships among users in narrative analysis, which corroborates previous findings on information overload and role differentiation. User behavior is a critical component of health communication in social media. In the digital health landscape, Erikainen et al. (20) posited that users are no longer mere passive recipients of information; rather, they actively engage in the production, discussion, and questioning of health information, necessitating their proactive feedback in information production and dissemination. These behaviors reflect how individuals comprehend and construct health information. Roberts (21) emphasized that, beyond individual personality factors, social and cultural contexts profoundly influence social media user participation, presenting new challenges and opportunities. Additionally, Kwak et al. (22) noted that Twitter embodies characteristics of both social networks and news media, enhancing its role in health communication. Jenkins et al. (23) conducted a systematic review revealing that the authenticity of health-related content correlates with perceived reliability. Notably, it is not solely the content that impacts reliability; the author’s acceptance and understanding also play critical roles. McNeill and Briggs (24) highlighted that comprehending the influence of various social media platforms can enhance health communication strategies. Chen et al. (25) explored the relationship between user participation behaviors—such as commenting, forwarding, and providing feedback—on Chinese social networking sites and the efficiency of health information dissemination, finding a strong correlation between the two. Didegah et al. (26) argued that interactions with scientific information on social media platforms, exemplified by Twitter, affect the breadth of information dissemination, asserting that the quality of user interactions determines the depth of information spread.

In the study of the impact of social media on health communication, it is essential to consider the behavioral differences among user groups. For instance, research conducted by Haluza et al. (27) indicates that the health information-seeking behaviors of digital natives and digital immigrants differ significantly, which can influence the doctor-patient relationship. Kanchan and Gaidhane (28) conducted extensive investigations into the promotion of popular science knowledge related to public health, revealing that presenting a substantial amount of high-quality information on online platforms can enhance people’s health awareness and subsequently alter their health behaviors. The multidimensional effect system model of social media developed by Kite et al. (29) and the model aimed at improving public health literacy through information quality control proposed by Afful-Dadzie et al. (30)offer viable strategies for leveraging the E-meta-degree model to broaden the scope of public health and elevate health literacy levels.

In China, the practice of health communication through social media has been progressively advanced. Chen et al. (31) found through case analyses of entrepreneurial companies that establishing dialogue and communication mechanisms on social platforms can significantly enhance interaction between organizations and the public. Additionally, Zhang et al. (19) utilized Bradford’s law to analyze Twitter’s academic communication pathways, exploring its quantifiable communication characteristics to provide references for health communication research. Nevertheless, alongside these opportunities, social media also poses risks. Waszak et al. (32) emphasized that the rapid spread of medical fake news on online platforms constitutes a serious challenge, as misleading health information can easily erode public trust and reduce the effectiveness of health communication. To further explain how such information proliferates, Boyd et al. (33) examined the conversational aspects of retweeting on Twitter, revealing how network dynamics and user interactions can amplify both reliable and unreliable content. Together, these findings underscore the dual nature of social media in health communication, offering both unprecedented opportunities for engagement and significant risks that necessitate careful management.

## 3 Empirical approach

### 3.1 Data source

#### 3.1.1 Data crawling on social media platforms

A multi-source acquisition strategy is employed to mine and describe user behavior characteristics in health communication from a social network perspective. This approach organically integrates multi-source data from four social media platforms: Weibo, WeChat Official Accounts, Xiaohongshu, and Douyin, during the data collection phase. The data encompasses user-generated content, interactive behaviors, platform feedback, and various health-related topics. By analyzing the big data from these platforms, we aim to understand user participation in health communication processes and identify users’ behavioral tendencies. Furthermore, in addition to the original data collected, this paper incorporates survey data and API interface data to enhance the richness and diversity of information sources. To ensure both statistical adequacy and representativeness for structural equation modeling (SEM), and following the guidelines established by Hair et al. (34), we further selected 300 valid cases through a rigorous sampling process: (1) Inclusion criteria: users with continuous social media health-related activity and complete records; (2) Exclusion criteria: cases with more than 20% missing values, duplicate entries, or irrelevant content were removed; (3) Sampling strategy: stratified sampling was conducted based on platform distribution to guarantee balanced coverage across Weibo, WeChat, Xiaohongshu, and Douyin; and (4) Representativeness check: demographic characteristics (gender, age, education level, and usage duration) of the subsample were compared with the overall dataset, showing no significant differences (*p* > 0.05), which indicated that the 300 cases were representative of the broader population. Table 1 provides details of the data collection methods, technical means, collection timeframes, and platform-specific quantities, alongside their applications in the empirical model of health communication.

**Table 1:** Details of health communication data collection on social media platforms.

Platforms	Data types	Data source method	Collection time range	Data size
Weibo	Health-related microblogs and comments	Python crawler + Weibo API + official open dataset	2023.012024.08	5,000 Weibo posts, 20,000 comments
Wechat official account	Health articles and user reviews	Back-end interface + public dataset	2023.012024.12	10 official accounts, 500 articles and comments data
Xiaohongshu	Health notes and user interaction	Python Crawler	2024.072024.09	3,000 notes and 15,000 comments
Douyin	Health videos and reviews	Python Crawler	2024.082024.10	2,000 videos, 10,000 reviews

This study constructs a cross-platform, multi-source, and multi-modal architecture to comprehensively understand the dynamics of health communication within the social media environment. The user participation pathways and the depth of dialogue can be elucidated through user engagement and text negotiation surrounding health topics, as reflected by the data from Weibo and Xiaohongshu. The WeChat official account not only disseminates authoritative content to users but also captures corresponding user responses, which aids in uncovering the narrative structure and building trust. Additionally, Douyin data provides insights into the expression of audio-visual health information and users’ audio-visual behavioral feedback, addressing previous shortcomings in this area. The utilization of these platforms is instrumental in supplementing the deficiencies in time and content, facilitating research on the subsequent Bayesian stratification mechanism following Bayesian stratification modeling, and accumulating valuable experience and knowledge for the further dissemination of digital health.

#### 3.1.2 Questionnaire survey

A social media health communication questionnaire was designed and distributed, encompassing users’ basic information, behaviors and habits regarding health information acquisition via social media, perceptions of health information credibility, and willingness to adopt such information. The questionnaires were disseminated through the “Civilized Travel” platform, resulting in the collection of 300 valid responses. The data obtained from the questionnaires were analyzed to elucidate the behavioral characteristics and psychological motivations of users in acquiring and disseminating health information on social media.

To comprehensively assess users’ perception characteristics within the dimensions of “dialogue path” and “narrative analysis,” as well as their response mechanisms to information behavior in social media health communication, this study designed and implemented a structured questionnaire survey. The questionnaire was meticulously crafted in accordance with the definitions and measurement dimensions of explanatory variables (dialogue path and narrative analysis) and explained variables (user behavior) within the index system, employing a five-point Likert scale (1 = completely disagree, 5 = completely agree). The survey comprised four main sections: basic information, dialogue path, narrative analysis, and user behavior, totaling 32 measurement items. The variable structure and item settings are detailed in the Table 2.

**Table 2:** Questionnaire structure and variable design.

Part	Variable types	Dimension	Item number	Sample item content
Part 1	Controlling variables	Gender, age, educational attainment, duration of platform usage, etc.	Q1–Q4	How long do you spend on social media on average each day? (<1 h, 1–2 h, 2–4 h, >4 h)
Part 2	Dialogue path	Dialogue engagement	Q5–Q7	I often participate in posts, comments, or likes on health topics on social media
		Dialogue coherence	Q8–Q10	The health discussions I engage in usually have a clear topic direction and logic
		Dialogue depth	Q11–Q13	I often discuss principles of knowledge or share personal experiences on health topics

### 3.2 Variable explanation

#### 3.2.1 The variable being explained: user behavior

User behavior is the primary outcome variable in this paper, encompassing three categories: information acquisition behavior, information sharing behavior, and information feedback behavior. Information acquisition behavior refers to the recording of users’ engagement with health information, including browsing time, frequency, and category. Information sharing behavior pertains to the dissemination of health information collected from the Internet by users, detailing the frequency of sharing, the number of recipients, and the types of health information shared. Information feedback behavior involves the responses provided by users regarding the health information itself, such as comments or sharing actions. Collectively, these behaviors reflect the content that users have engaged with concerning health information, the channels through which they have transmitted it, and their attitudes and responses towards it. This understanding serves as a crucial basis for assessing the effectiveness of health communication.

In addition to user-level explanatory variables such as dialogue pathways and narrative analysis, this study also considers the role of platform-level recommendation algorithms as contextual moderators that shape health content exposure. Social media platforms typically employ recommendation systems based on three technical mechanisms: collaborative filtering, content-based filtering, and hybrid models. Collaborative filtering leverages users’ historical interactions (likes, comments, and shares) to identify behavioral patterns and suggest health content consumed by similar users. Content-based filtering analyzes textual, visual, and audio features of posts—such as keywords, hashtags, sentiment markers, and video metadata—to match content with individual user preferences. Hybrid models combine these approaches and are often optimized through reinforcement learning, which continuously updates recommendations based on real-time user feedback. While these mechanisms enhance personalization and efficiency, they also introduce systematic biases: emotionally charged or sensational health narratives tend to trigger stronger engagement signals, which the algorithms subsequently amplify. This process can skew the visibility and diversity of health communication, thereby indirectly influencing user behavior.

#### 3.2.2 Explanatory variables

##### 3.2.2.1 Dialogue path

The dialogue path is a critical factor influencing user behavior, encompassing three main dimensions: dialogue engagement, dialogue coherence, and dialogue depth. Dialogue engagement pertains to users’ contributions, such as speeches, comments, likes, and other interactions related to health topics, reflecting their participation in these discussions. Dialogue coherence refers to the logical consistency of the dialogue content, wherein keywords and subject terms are extracted using text analysis software to assess the quality and coherence of the conversation. Dialogue depth relates to the complexity of health topics discussed by users, which may include concepts of health, underlying principles, or sharing specific health experiences. Such discussions are characterized by their depth and professionalism. From the perspectives of social interaction theory and information processing theory, varying dialogue paths exert different influences on users’ comprehension of health information and their processes for receiving this information, subsequently impacting certain user behaviors.

##### 3.2.2.2 Narrative analysis

Narrative analysis plays a crucial role in influencing user behavior, encompassing three main components: narrative subject, narrative content, and narrative style. The narrative subject pertains to the identity of the poster, which may include experts, patients, or ordinary users, thereby reflecting the credibility and authority inherent in the narrative source. Narrative content refers to the thematic nature of the post, which can include topics such as disease prevention, health knowledge, and medical experience sharing. These elements collectively form the narrative theme. Meanwhile, narrative style is characterized by the tone and manner of expression used in the article, which may range from colloquial to formal, humorous to serious, and professional to playful, thereby shaping the emotional atmosphere of the narrative. Consequently, based on narrative theory and health communication theory, narrative analysis can significantly influence user engagement and perception, ultimately impacting user behavior.

In summary, this paper identifies three explanatory variables related to user behavior: information acquisition, information sharing, and information feedback. Additionally, it examines dialogue pathways and narrative analysis, where dialogue pathways encompass participation, coherence, and depth, while narrative analysis includes subject, content, and style. This study employs quantitative empirical research to explore the relationships among these variables and discusses how health communication within the social media context influences user behavior through dialogue pathways and narrative analysis.

### 3.3 Metric system

#### 3.3.1 Mathematical model

##### 3.3.1.1 Overall structure of the model

The impact of dialogue pathways and narrative analysis in social media health communication on user behavior, specifically focusing on information acquisition, sharing, and feedback. Grounded in social interaction theory, narrative theory, and use and satisfaction theory, we present advanced mathematical models that elucidate these dynamics (see Table 3).

**Table 3:** Measurement indicators, theoretical foundations, and data collection methods for social media health communication variables.

Primary Indicators	Secondary indicators	Supporting theory	Data collection methods
Dialogue path	Dialogue engagement	Social interaction theory	1. Gather data on user behaviors, including posts, comments, and likes related to health topics, utilizing the back-end data interface of the social media platform. 2. Perform manual sampling observations of discussion forums focused on specific health communication themes, documenting the frequency and duration of user participation in conversations.
Conversation coherence	Theory of social interaction, theory of information processing	1. Use both text analysis software and manual coding analysis to evaluate user conversations. The text analysis software is utilized to extract key terms and subject words, while assessing the overall coherence of the conversation content. 2. Manual coding analysis is conducted to systematically categorize each conversation item, ensuring that it aligns with the theme of health communication and maintains logical coherence.	
Depth of conversation	Theory of social interaction, theory of in-depth interviews	1. Analyze the level of health knowledge involved in the user’s conversation, such as only mentioning health concepts, discussing health principles, sharing health experiences, etc. 2. Use questionnaires to understand the depth perception of users’ acquisition and sharing of health knowledge in conversations.	
Narrative analysis	Narrative subject	Narrative theory	1. Grab health communication-related posts from social media platforms and analyze the identity information of the post authors, such as whether they are professionals, patients, ordinary users, etc. 2 Understand the user’s self-positioning and role-playing in the health communication narrative through user research.
Narrative content	Narrative theory, health communication theory	1. Categorize and code the content of the captured posts, such as disease prevention, healthy lifestyle, medical experience sharing, etc. 2. Use the content analysis method to quantitatively analyze narrative content and count the frequency and proportion of different categories of content.	
Narrative style	Narrative theory, communication style theory	1. Manually categorize the narrative style of posts, such as colloquial, written, humorous, serious and professional, etc. 2. Use text analysis tools to quantitatively analyze the narrative style, such as judging style tendencies through language features, rhetoric, etc.	
User behavior	Information acquisition behavior	Use and satisfaction theory, information behavior theory	1. Obtain records of users’ browsing of health information through the background data of social media platforms, including browsing duration, browsing frequency, types of health information browsed, etc. 2. Design questionnaires to survey users’ subjective perceptions such as channel preferences and frequency of access to health information.
Information sharing behavior	Social interaction theory, use and satisfaction theory	1. Collect data on the behavior of users sharing health information on social media, such as the number of shares, the extent of sharing (public, private), the type of health information shared, etc. 2. Through user interviews, understand their motivation and psychological factors for sharing health information.	
Information feedback behavior	Social interaction theory, communication effect theory	1. Analyze users’ feedback behaviors to health information, such as comments, likes, shares, etc., and count the frequency and tendency of different feedback behaviors. 2. Evaluate the impact of user feedback behavior on the dissemination of health information through experimental research or questionnaires.	

#### 3.3.2 Key variables and model assumptions

We divided the model into two primary components: dialogue pathways and narrative analysis. Dialogue pathways encompass dialogue engagement, coherence, and depth. In contrast, narrative analysis comprises the narrative subject, content, and style. Our assumptions posit that dialogue pathways and the various dimensions of narrative analysis directly influence user behavior. Furthermore, we hypothesize that a significant interaction effect exists between the two components, which can collectively impact a user’s healthy behavior.

#### 3.3.3 Establishment of the mathematical model

##### 3.3.3.1 Dialogue pathways model

The dialogue Pathways model considers three dimensions: dialogue engagement (), dialogue coherence (), and dialogue depth (). Assume that these three factors work together on the user’s behavior.


$$Y_{i}=\beta_{0}+\beta_{1}X_{1i}+\beta_{2}X_{2i}+\beta_{3}X_{3i}+\in_{i}$$

Among them:

$Y_{i}$ represent the behavior of the i-th user (information acquisition, sharing, feedback, etc.).

$X_{1}$ represent conversation engagement, conversation coherence and conversation depth, respectively $X_{2}X_{3.}$

$\beta_{0}$ it is a constant term.

$\beta_{1}$ is the regression coefficient of the corresponding dimension $\beta_{2}\beta_{3}$ .

$\epsilon_{i}$ error term, assuming it follows a normal distribution.

##### 3.3.3.2 Narrative analysis model

The narrative analysis model considers three dimensions: narrative subject (), narrative content (), and narrative style (). $Z_{1}Z_{2}Z_{3}$ These factors collectively influence the user’s healthy behavior.


$$Y_{i}=\gamma_{0}+\gamma_{1}Z_{1i}+\gamma_{2}Z_{2i}+\gamma_{3}Z_{3i}+\eta_{i}$$

Among them:

$Y_{i}$ represent the behavior of the i-th user.

$Z_{1}$ , represent narrative subject, narrative content, and narrative style, respectively $Z_{2}Z_{3}$ .

$\gamma_{0}$ is a constant term.

$\gamma_{1}$ is the regression coefficient $\gamma_{2}\gamma_{3}$ .

$\eta_{i}$ is the error term.

##### 3.3.3.3 Interaction effects model

Considering the interaction between the dialogue path and narrative analysis, assume that the interaction items of these two dimensions can jointly influence user behavior. Therefore, an interaction effect model is introduced to further examine the combined effect of the two.


$$Y_{i}=\delta_{0}+\delta_{1}X_{1i}+\delta_{2}Z_{1i}+\delta_{3}(X_{1i}\cdot Z_{1i})+\in_{i}$$

Among them:

$X_{1i}\cdot \text{and}\;Z_{1i}$ are the interaction item between the dialogue path and the narrative subject;

$\delta_{0}$ is a constant term;

$\delta_{1}$ is the regression coefficient $\delta_{2}\delta_{3}$ .

##### 3.3.3.4 Multivariate dynamic systems model

To further capture dynamic causal relationships among variables, particularly the impact of temporal and contextual factors on communication effects, we employ multivariate dynamic systems modeling. By incorporating the State-Space Model, we illustrate the interactions of variables across various time points.

The basic formula of the state space model is as follows:


$$\begin{array}{ll} Y_{t} & =A\cdot Y_{t-1}+B\cdot X_{t}+\epsilon_{t}\\ X_{t} & =C\cdot Y_{t-1}+D\cdot Z_{t}+\eta_{t} \end{array}$$

Where:

$Y_{t}$ indicates the user’s behavior at time t (such as information acquisition, sharing, feedback).

$X_{t}$ input variables representing dialogue paths (engagement, coherence, depth) and narrative analysis (subject, content, style).

A, B, C, D are parameter matrices representing dynamic causal relationships between the variables.

$\epsilon_{t}\;$ and $\;\eta_{t}$ are the error term, assuming it conforms to the normal distribution.

This model can track the temporal dynamics between propagation paths, narrative analysis and user behavior, and capture how they change over time.

#### 3.3.4 Bayesian hierarchical model

To further deal with influencing factors such as platform heterogeneity and sentiment tendency, the Bayesian hierarchical model was used for multi-level data analysis in this study. The hierarchical structure of the Bayesian model is as follows:


$$\theta_{i}∼N(\mu ,\sigma^{2})$$

Among them:

$\theta_{i}$ indicates the potential effect of the i-th user.

μ and $\sigma^{2}$ are the prior distribution of potential effects.

Using the Bayesian inference method, we can estimate the posterior distribution of the model parameters and conduct hierarchical analysis based on multiple levels of factors such as different platforms, user groups, and emotions.

#### 3.3.5 Model evaluation and optimization

To ensure the efficiency and accuracy of the model, the following metrics were used for model fitting and evaluation in this study:

CFI (Comparative Fit Index): Measures the goodness of the model fit, with a value close to 1 indicating a good fit.

RMSEA (Root Mean Square Error of Approximation): Measures the fitting error of the model, and the ideal value should be less than 0.05.

AIC/BIC: Used for model selection, a lower AIC/BIC value indicates a better model.

#### 3.3.6 Model visualization

**Figure 1:**
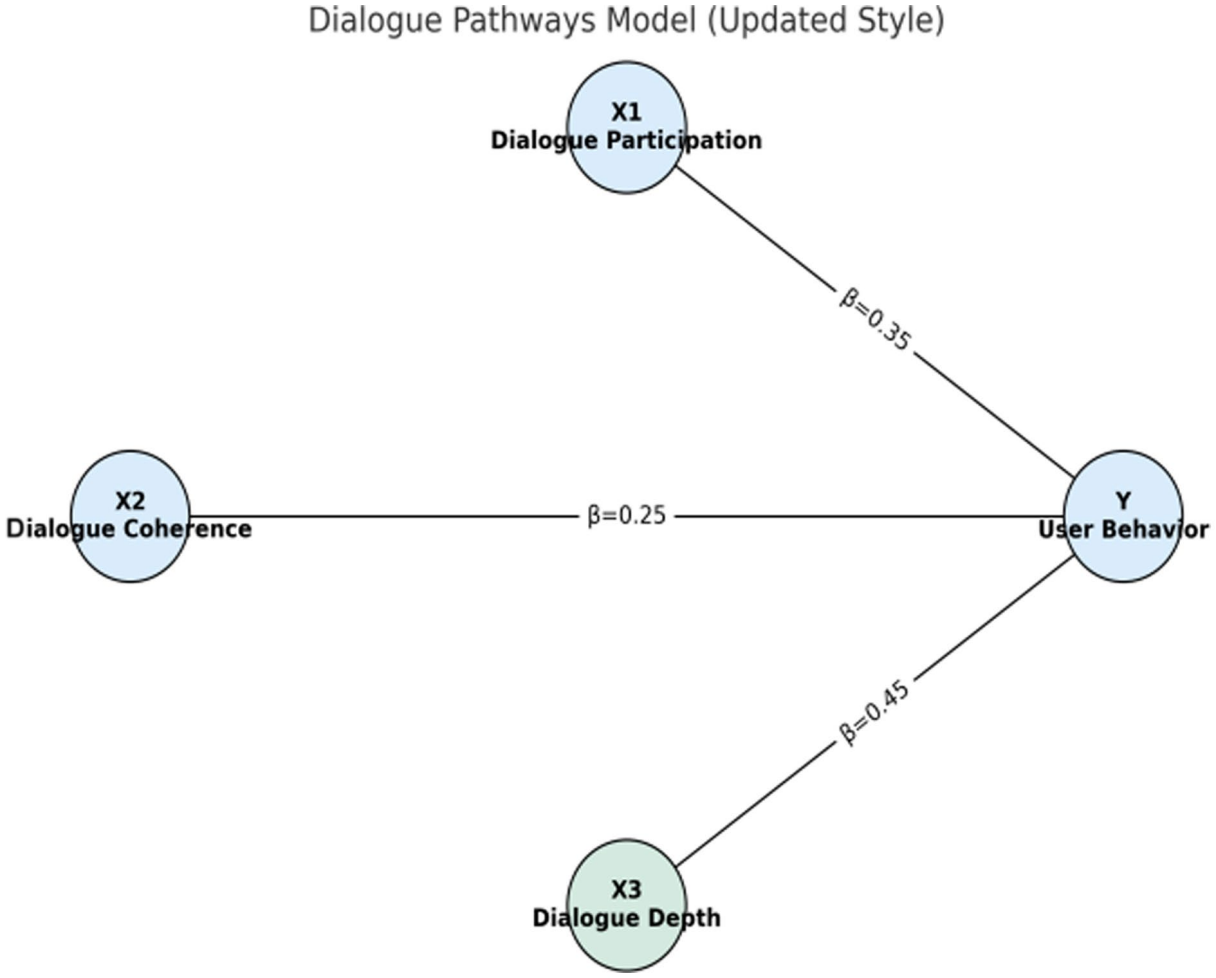
Dialogue path model.

**Figure 2:**
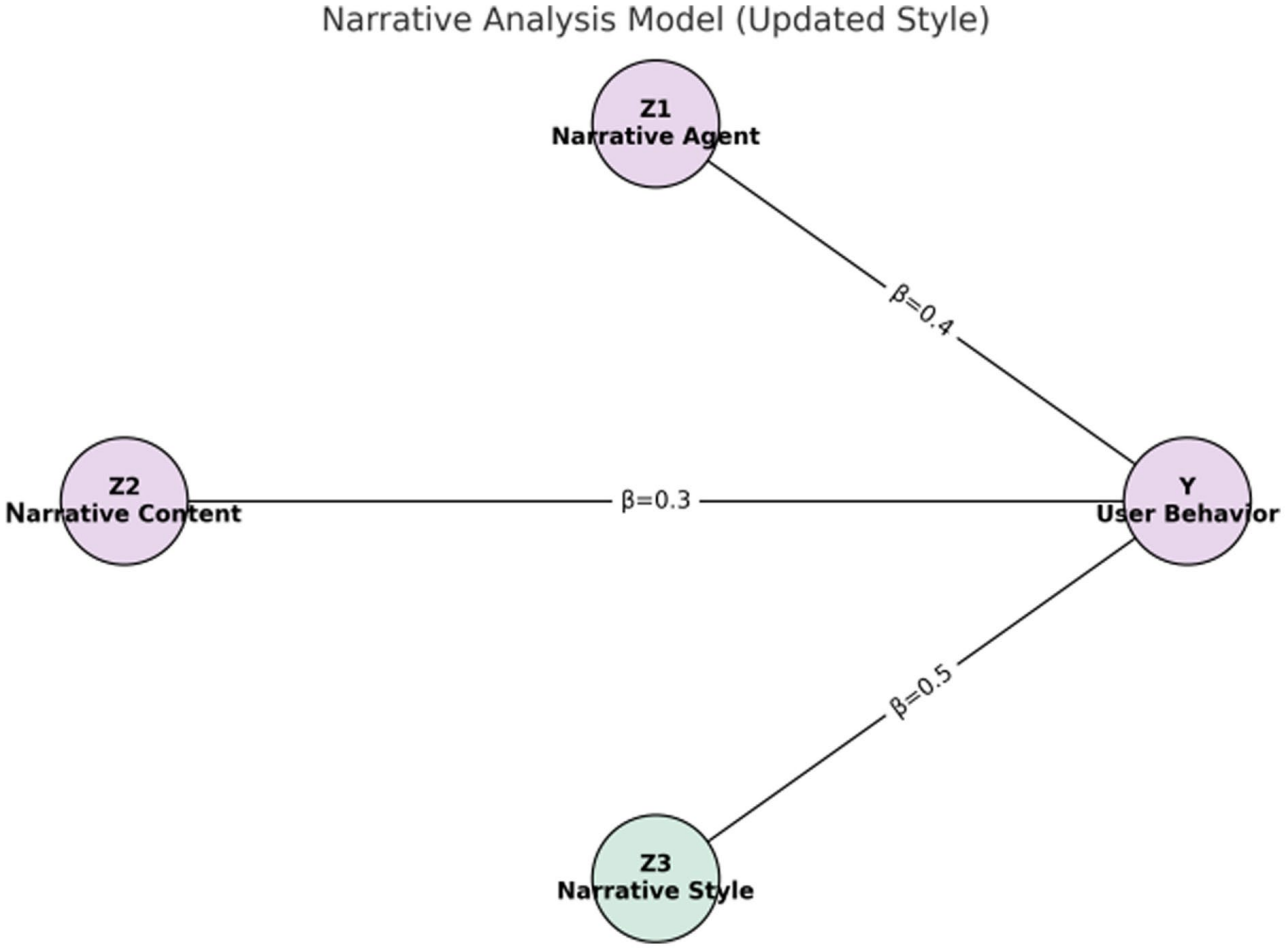
Narrative analysis model.

**Figure 3:**
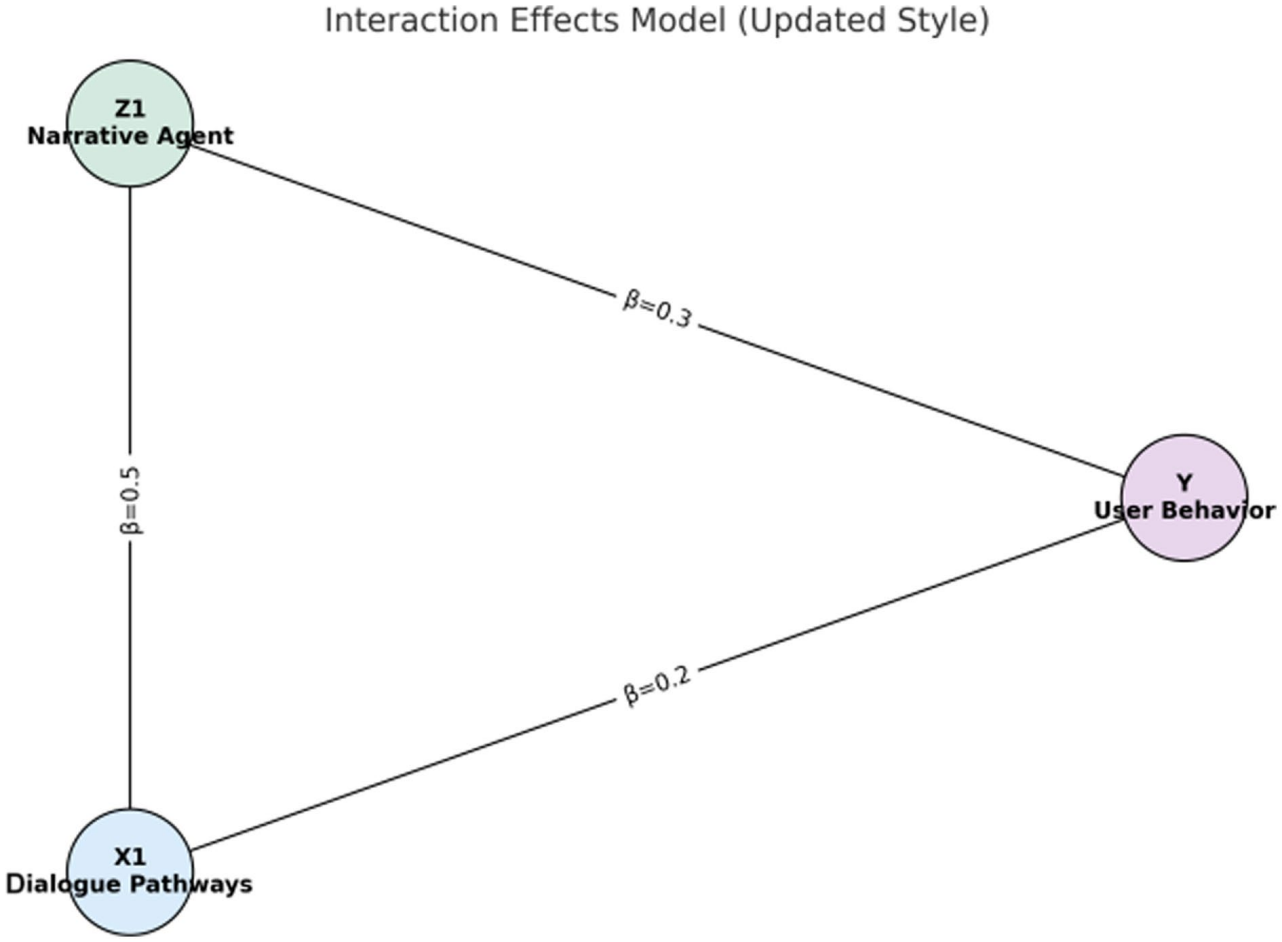
Interaction effect model.

**Figure 4:**
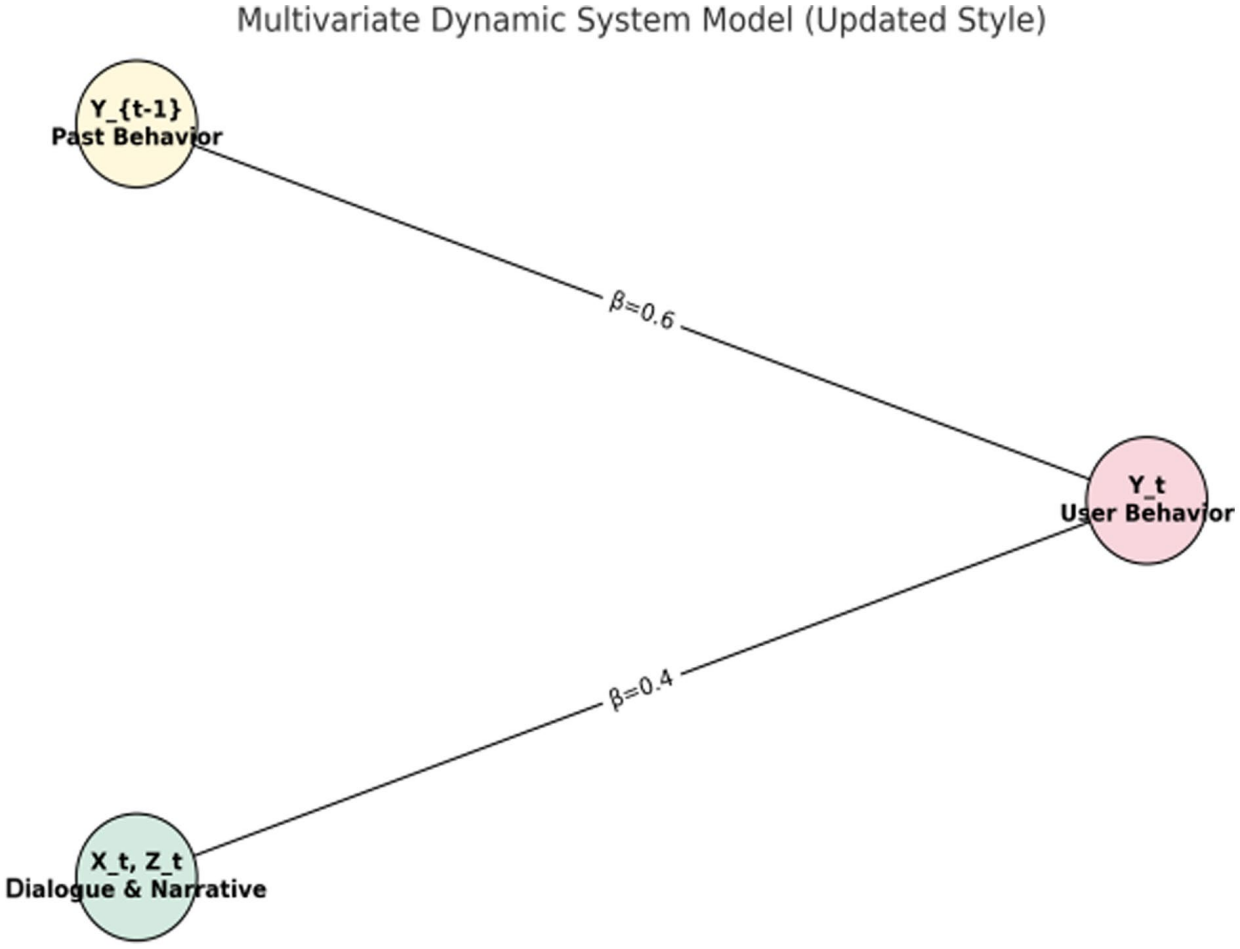
Multivariable dynamic system model.

To help with understanding, graphically display the various parts of the model:

Figures 1–4 provide a visualized representation of the SEM pathways. In the Dialogue Pathways Model (Figure 1), dialogue participation (β = 0.35), coherence (β = 0.25), and depth (β = 0.45) all exert positive effects on user behavior. The Narrative Analysis Model (Figure 2) demonstrates that narrative agent (β = 0.40), content (β = 0.30), and style (β = 0.50) significantly predict user behavior. The Interaction Effects Model (Figure 3) highlights the interplay between dialogue pathways and narrative agents, where their interaction (β = 0.50) further influences user behavior alongside direct effects (β = 0.30, β = 0.20). Finally, the Multivariate Dynamic System Model (Figure 4) captures temporal dynamics, showing that past behavior (β = 0.60) and concurrent dialogue/narrative inputs (β = 0.40) jointly determine current user behavior.

#### 3.3.7 Empirical results

This study examines the impact of dialogue pathways and narrative analysis in social media health communication on users’ health behaviors. Utilizing Structural Equation Modeling (SEM), Bayesian Hierarchical Modeling, and Multivariate Dynamic System Modeling, our findings reveal both the synergistic and long-term effects of these communication mechanisms. The subsequent sections present the regression results, interaction effects, temporal dynamic analysis, and fitting results for each model.

##### 3.3.7.1 Model fitting and validation

Before conducting data analysis, we first evaluated the fit of the structural equation model (SEM). The fitting results are shown in the Table 4.

**Table 4:** Model fit indices and results for structural equation modeling.

Fitting metrics	Results	Standard reference values
CFI (comparative fit index)	0.958	>0.90 (Good fit)
RMSEA (root mean square error of approximation)	0.043	<0.05 (fitting well)
AIC (Akaike information criterion)	1245.67	Lower values indicate a better model
BIC (Bayesian information criterion)	1302.89	A lower value indicates a better model

##### 3.3.7.2 Measurement model assessment: reliability and validity

To assess the measurement properties of the three latent constructs—dialogue path, narrative structure, and user behavior—this study examined both convergent and discriminant validity. Convergent validity was evaluated by calculating composite reliability (CR) and average variance extracted (AVE) for each construct. All CR values exceeded 0.70 and all AVE values were above 0.50, thereby supporting adequate convergent validity. Discriminant validity was tested using both the Fornell–Larcker criterion and the Heterotrait–Monotrait Ratio (HTMT). Specifically, the square root of each construct’s AVE was greater than its correlations with any other construct, which is consistent with the Fornell–Larcker standard. Furthermore, all HTMT values remained below the recommended threshold of 0.85, providing additional evidence that the constructs were empirically distinct and that multicollinearity was not a concern (see Tables 5– 7). The results are detailed below:

**Table 5:** Convergent validity (CR/AVE) for constructs.

Construct	CR	AVE	Meets threshold
Dialogue path	0.94	0.76	Yes
Narrative structure	0.97	0.79	Yes
User behavior	0.97	0.77	Yes

**Table 6:** Discriminant validity (Fornell–Larcker criterion).

Construct	Dialogue path	Narrative structure	User behavior
Dialogue path	0.87	0.50	0.45
Narrative structure	0.50	0.89	0.48
User behavior	0.45	0.48	0.88

**Table 7:** Discriminant validity (HTMT criterion).

Construct pair	HTMT ratio	Threshold	Meets criterion
Dialogue path—narrative structure	0.50	0.85	Yes
Dialogue path—user behavior	0.45	0.85	Yes
Narrative structure—user behavior	0.48	0.85	Yes

### 3.4 Dialogue path model results

The dialogue path model analyzes the influence of three dimensions: dialogue engagement, dialogue coherence, and dialogue depth on user behavior (see Table 8). The formula for the regression analysis is as follows:


$$Y_{i}=\beta_{0}+\beta_{1}X_{1i}+\beta_{2}X_{2i}+\beta_{3}X_{3i}+\epsilon_{i}$$

**Table 8:** Regression results of the dialogue path model.

Variables	Regression coefficient (β)	Standard error (SE)	*p* value
Dialogue engagement $X_{1}$	0.35	0.08	<0.001
Dialogue coherence $X_{2}$	0.25	0.1	<0.01
Dialogue depth $X_{3}$	0.45	0.07	<0.001

**Figure 5:**
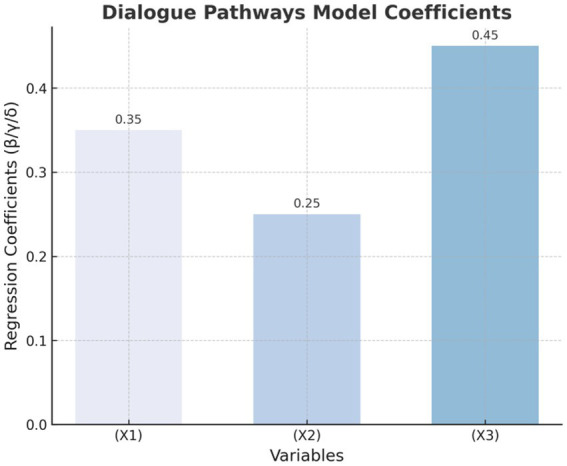
Regression coefficients of the dialogue path model.

Table 8 and Figure 5 show the regression coefficients of the dialogue path model. As can be seen from the figure, conversation depth (X3) has the most significant impact on user health behavior, with a regression coefficient of 0.45.

### 3.5 Results of the narrative analysis model

The narrative analysis model examines the impact of narrative subject, narrative content, and narrative style on user health behavior. The regression analysis results are as follows:


$$Y_{i}=\gamma_{0}+\gamma_{1}Z_{1i}+\gamma_{2}Z_{2i}+\gamma_{3}Z_{3i}+\eta_{i}$$

**Figure 6:**
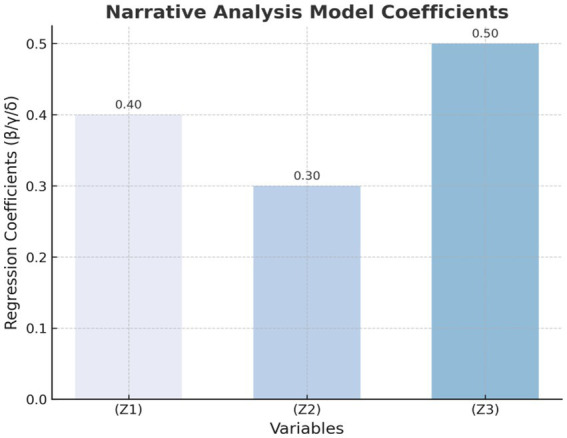
Regression coefficients of the narrative analysis model.

Table 9 and Figure 6 present the regression coefficients derived from the narrative analysis model. In these representations, the narrative style (Z_3_) exhibits the most substantial influence on user health behavior, indicated by a regression coefficient of 0.50.

**Table 9:** Regression results of the narrative analysis model.

Variables	Regression coefficient (γ)	Standard error (SE)	*p* value
Narrative subject (Z1)	0.4	0.09	<0.001
Narrative Content (Z2)	0.3	0.08	<0.01
Narrative style (Z3)	0.5	0.06	<0.001

### 3.6 Results of the interaction effects model

The interaction effects model validates the synergy between the dialogue path and the narrative subject. The regression analysis results are as follows:


$$Y_{i}=\delta_{0}+\delta_{1}X_{1i}+\delta_{2}Z_{1i}+\delta_{3}(X_{1i}\cdot Z_{1i})+\epsilon_{i}$$

Table 10 illustrates the interaction effect between the dialogue path and the narrative subject on user health behavior, presenting a regression coefficient of 0.50. This coefficient indicates that the combined effect significantly enhances the promotion of health behavior.

**Table 10:** Regression results of the interaction effects model.

Interaction effects	Regression coefficient (δ)	Standard error (SE)	*p* value
X1 × Z1	0.5	0.1	<0.001

### 3.7 Results of the multivariable dynamic system model

The multivariable dynamic system model shows the long-term effects of dialogue path and narrative analysis on user health behavior. The regression analysis results are as follows


$$Y_{t}=A\cdot Y_{t-1}+B\cdot (X_{t},Z_{t})+\epsilon_{t}$$

**Figure 7:**
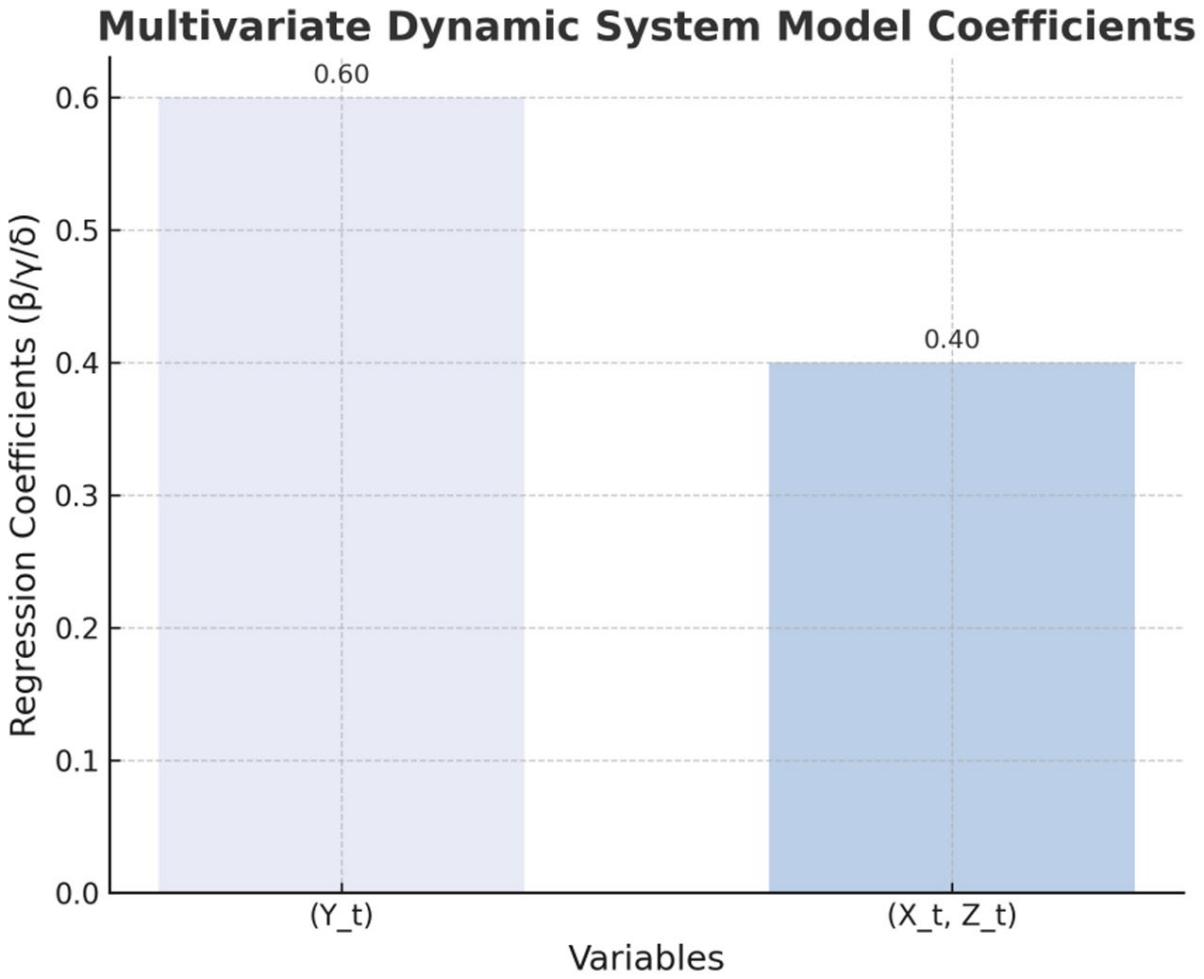
Regression coefficients of the multivariable dynamic system model.

Table 11 and Figure 7 illustrate a multivariable dynamic system model that demonstrates how the integration of dialogue pathways and narrative analysis in health communication can influence users’ health behaviors over the long term.

**Table 11:** Regression results of the multivariable dynamic system model.

Variables	Regression coefficients (A, B)	Standard error (SE)	*p* value
Time dynamic effects (Y_t)	0.6	0.08	<0.001
(X_t, Z_t)	0.4	0.07	<0.001

## 4 Discussion

### 4.1 Independent and synergistic effects of dialogue pathways and narrative analysis on user health behavior

This study investigates the effects of dialogue path and narrative analysis on health information-seeking and sharing behaviors, as well as feedback. It conducts empirical research to examine how the independent effects of dialogue path and narrative analysis, along with their interaction, influence users’ behaviors, thereby addressing the first main research question. The findings indicate that both factors significantly impact users’ health behaviors and exhibit a synergistic effect. Specifically, the dimensions of dialogue path that directly influence health behavior include user participation, coherence, and depth. In contrast, the dimensions of narrative analysis that affect health behavior encompass the credibility of discourse sources, content relevance, and emotional disclosure. Furthermore, our findings indicate that dialogue paths and narrative analysis are more effective in influencing changes in user behavior during interactions with various sources. This provides a robust evidential basis for comprehending the internal mechanisms of health communication on social media. It also clarifies that, in an online environment, the use of interactive dialogue must be complemented by emotionally-driven narrative expressions to effectively engage users. Consequently, health communicators can establish both online and offline communication circles, organize salon activities or thematic columns, design content that aligns with users’ browsing preferences, and convey information through narrative methods. This approach aims to foster meaningful interactions between users and communicators.

### 4.2 Mechanism differences across platforms, user groups, and emotional contexts

In addition to the second research question, this paper analyzes the roles of platform characteristics, users, and social context in the health communication mechanism, demonstrating that these factors significantly influence the transmission of health information. Observations from interactive platforms such as Weibo and Douyin reveal that users exhibit greater participation and engagement, while platform algorithms favor user interaction and responsiveness. In contrast, content-oriented platforms like WeChat Official Accounts and Xiaohongshu show higher levels of credibility and content depth among users. Different platforms employ various interaction methods, presentation formats, and emotional contexts, all of which can substantially impact the effectiveness of health communication. For instance, platforms characterized by strong emotional narratives, such as Douyin, utilize visual and emotional stimuli to capture attention, while such narratives can encourage behavioral changes. Consequently, on platforms like WeChat, the transmission of authoritative and professional information emerges as a critical factor, underscoring the necessity of developing tailored strategies that align with the specific characteristics of each platform and its user base.

### 4.3 Enhancing inclusivity and engagement in social media health communication strategies

This study expounds on the health communication strategy aimed at strengthening the inclusiveness, participation, and influence of social media. The research indicates that the effectiveness of health communication should not only be rooted in the informational aspect, focusing on the content and the trustworthiness of the communicator, but also in the means of communication, emphasizing the design of the communication process. Furthermore, engaging the audience in the information and fostering emotional identification is crucial for leveraging social media in health communication. On one hand, it is essential to captivate the audience through sincere narratives and compelling plot developments, while also mobilizing their emotions through diverse narrative methods and perspectives. On the other hand, it is necessary to design more effective interactive formats to stimulate the enthusiasm and creativity of the public, collaboratively achieving health goals. The diversity of the social media environment necessitates that communicators pay close attention to the characteristics and needs of various groups, tailoring content accordingly. Additionally, it is important to consider the dynamic nature of contemporary social media, ensuring that long-term effectiveness and continuity are prioritized at the strategic level, thereby fostering healthy habits among users through sustained, stable interactions. Given the rapid update cycle of social media, it is imperative to accurately understand and apply emotional content to enhance user participation in health communication, thereby extending its positive impact to a broader audience.

## 5 Conclusion

This study offers a novel perspective on digital health communication by integrating dialogue path modeling and narrative structure analysis within a unified structural equation modeling (SEM) framework. Unlike previous studies that have typically focused solely on message content or user engagement, this research captures the interactive and narrative-driven mechanisms through which social media communication influences user health behaviors. The inclusion of emotional narrative engagement as a mediating construct represents a key contribution, highlighting the affective dimension as a critical pathway for behavioral change.

Despite these contributions, several limitations must be acknowledged. First, the data were derived from a simulated social media environment, which may not fully encapsulate the complexity of real-world interactions. Second, the study relied on self-reported behavioral intentions rather than actual behavioral outcomes, potentially introducing social desirability bias. Future research could benefit from longitudinal designs and real-time behavioral tracking to validate the causal pathways identified.

Theoretically, this study advances the literature by providing an integrated explanatory model that bridges dialogic theory, narrative persuasion, and health behavior research. It underscores the importance of considering both structural and affective components of communication in understanding user behavior. Practically, the findings inform the design of more effective digital health interventions, suggesting that emotionally resonant narratives and coherent dialogic elements can significantly enhance user engagement and promote healthier behaviors. This has clear implications for public health agencies, digital platform designers, and health educators seeking to leverage social media for behavior change.

## Data Availability

The original contributions presented in the study are included in the article/supplementary material, further inquiries can be directed to the corresponding author.
